# Hydrogen Peroxide-Preconditioned Human Adipose-Derived Stem Cells Enhance the Recovery of Oligodendrocyte-Like Cells after Oxidative Stress-Induced Damage

**DOI:** 10.3390/ijms21249513

**Published:** 2020-12-14

**Authors:** Patricia Garrido-Pascual, Ana Alonso-Varona, Begoña Castro, María Burón, Teodoro Palomares

**Affiliations:** 1 Department of Cell Biology and Histology, Faculty of Medicine and Nursing, University of the Basque Country (UPV/EHU), 48940 Leioa, Bizkaia, Spain; ana.alonsovarona@ehu.es (A.A.-V.); maria_buron@hotmail.com (M.B.); 2 Histocell, Bizkaia Science and Technology Park, 48160 Derio, Bizkaia, Spain; bcastro@histocell.com; 3 Department of Surgery, Radiology and Physical Medicine, Faculty of Medicine and Nursing, University of the Basque Country (UPV/EHU), 48940 Leioa, Bizkaia, Spain; teodoro.palomares@ehu.eus

**Keywords:** oxidative stress, human adipose-derived stem cells, oligodendrocyte-like cells, H_2_O_2_ preconditioning, antioxidant capacity, cell therapy

## Abstract

Oxidative stress associated with neuroinflammation is a key process involved in the pathophysiology of neurodegenerative diseases, and therefore, has been proposed as a crucial target for new therapies. Recently, the therapeutic potential of human adipose-derived stem cells (hASCs) has been investigated as a novel strategy for neuroprotection. These cells can be preconditioned by exposing them to mild stress in order to improve their response to oxidative stress. In this study, we evaluate the therapeutic potential of hASCs preconditioned with low doses of H_2_O_2_ (called HC016 cells) to overcome the deleterious effect of oxidative stress in an in vitro model of oligodendrocyte-like cells (HOGd), through two strategies: i, the culture of oxidized HOGd with HC016 cell-conditioned medium (CM), and ii, the indirect co-culture of oxidized HOGd with HC016 cells, which had or had not been exposed to oxidative stress. The results demonstrated that both strategies had reparative effects, oxidized HC016 cell co-culture being the one associated with the greatest recovery of the damaged HOGd, increasing their viability, reducing their intracellular reactive oxygen species levels and promoting their antioxidant capacity. Taken together, these findings support the view that HC016 cells, given their reparative capacity, might be considered an important breakthrough in cell-based therapies.

## 1. Introduction

Oxidative stress associated with neuroinflammation is a key process involved in the pathophysiology of neurodegenerative diseases such as Alzheimer’s, Parkinson’s and amyotrophic lateral sclerosis [1,2,3]. Although oxidative stress may affect any cell type in the body, the high oxygen consumption and the limited antioxidant capacity of nerve tissue makes neurons and glia especially vulnerable to increased ROS levels [4]. In particular, oligodendrocytes, the myelin-producing cells of the central nervous system (CNS), are notably vulnerable to oxidative stress due to their high metabolic rate and their low concentration of glutathione [5]. In a state of oxidative stress, oligodendrocytes may undergo irreversible damage of proteins, lipids and DNA, mitochondrial dysfunction, cell degeneration and death [6]. These types of damage could be attenuated thanks to cell therapy.

Today, the use of mesenchymal stem cells (MSCs) is a potential therapeutic tool for neurodegenerative diseases. This therapeutic proposal is based on the immunomodulatory and neuroprotective activity of MSCs, due to their antioxidant capacity and the release of anti-inflammatory cytokines, neurotrophic growth factors and components of the ECM [7,8]. Although it is possible to obtain MSCs from different niches, it has been shown that the secretion of neurotrophic growth factors is greater in human adipose-derived stem cells (hASCs) than in those derived from bone marrow [9], which may be due to the paracrine interrelation between adipose tissue and the CNS [10]. Nonetheless, like other types of MSCs, hASCs have low survival and engraftment rates after transplantation at the site of injury [11]. To address this problem, various cell preconditioning strategies have been tested [12,13,14]. The preconditioning process–sub-lethal exposure to cellular stressors–promotes the expression and secretion of certain molecules that are required to reduce damage and increase survival, giving cells the capacity to respond efficiently to a higher level of the same stressor [15,16,17]. In a previous study, we showed that hASCs preconditioned with low doses of H_2_O_2_ (HC016 cells) increase their resistance to oxidative stress by enhancing their intrinsic antioxidant capacity and bioenergetic regulation [18].

Therefore, in this study, we investigated whether HC016 cells, in addition to increasing their resistance and adaptation to harsh microenvironments, are able to improve their capacity to repair, in particular, oxidative damage in oligodendroglial cells. Herein, we established an in vitro model of oxidative stress-induced damage in oligodendrocyte-like cells and co-cultured them with HC016 cells and their conditioned media, in order to evaluate the therapeutic potential of HC016 cells and their derivates.

## 2. Results

### 2.1. HOG Cell Differentiation

**Figure 1:**
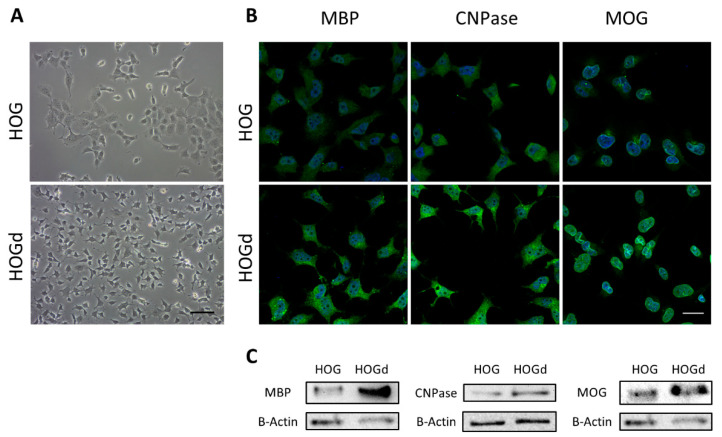
HOG cell line differentiation. (**A**) Phase-contrast images of HOG and HOGd cells. Differentiated cells showed short ramified processes (sale bar: 100 µm); (**B**) Representative images of the expression of MBP, CNPase and MOG evaluated by immunofluorescence (scale bar: 50 µm), and (**C**) Western blot. At least three different experiments were performed.

In this study, we differentiated HOG cells to oligodendrocyte-like cells (HOGd). Differentiation was assessed by analyzing their morphology and by quantifying MBP, CNPase and MOG expression by immunofluorescence and western blot analysis. HOGd exhibited extended branched processes and short membrane extensions (Figure 1A).

In addition, HOGd showed higher expression of MBP, CNPase and MOG than HOG (Figure 1B). MBP, the major structural component of myelin, was localized to the cytoplasmic surface of the plasma membrane as well as in the nucleus. CNPase, a membrane-associated enzyme in the myelin sheath, was also found on the cytoplasmic surface of the plasma membrane. Finally, MOG, a glycoprotein that is important in myelination, was detected around the nucleus. The higher expression of these proteins in HOGd showed by immunofluorescence was confirmed by western blot analysis (Figure 1C).

### 2.2. Capacity of HC016 Cells to Repair _OX_HOGd

Once the HOG cells had differentiated, they were exposed to 0.25 mM H_2_O_2_ for 1 h. The exposure of HOGd to H_2_O_2_ significantly increased intracellular ROS levels and reduced their proliferation rate compared to values in control cells. After the oxidation, the therapeutic potential of the HC016 cells was evaluated, both through their CM and indirect co-culture with the damaged HOGd (_OX_HOGd).

**Figure 2:**
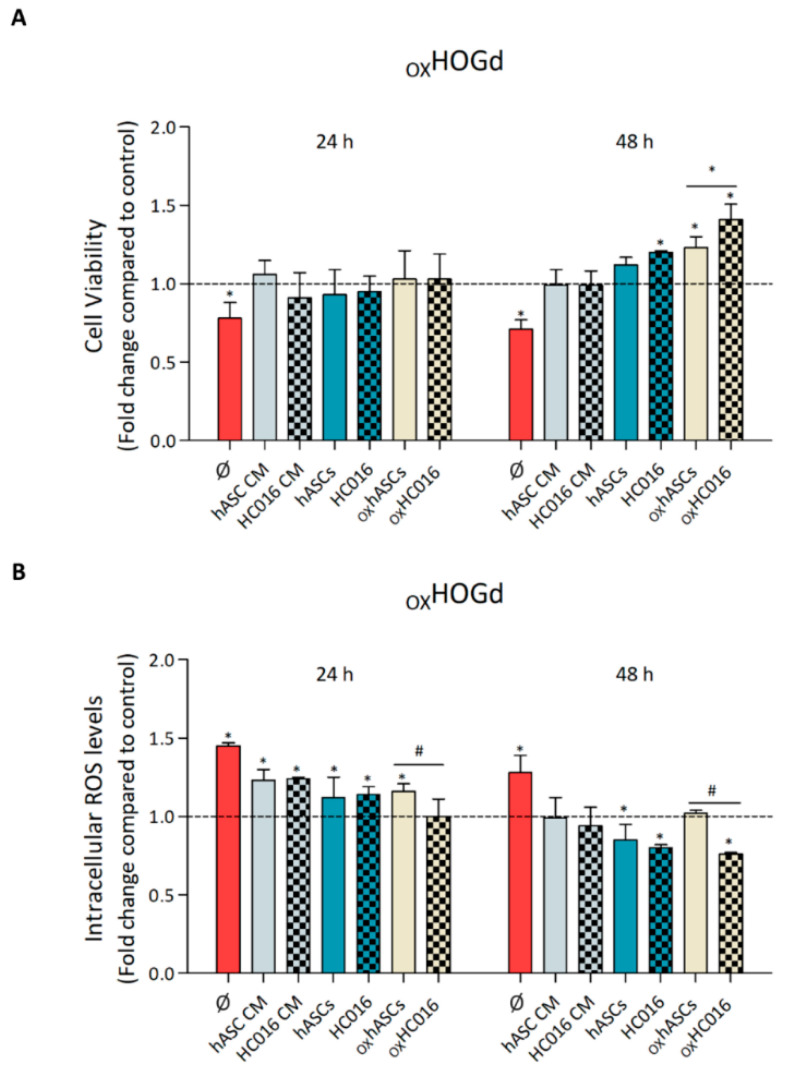
Reparative effect of hASCs and their derivatives on _OX_HOGd. (**A**) The viability and (**B**) intracellular ROS levels of _OX_HOGd were measured at different time points (24 and 48 h), alone (Ø) or cultured with: i, hASC CM or HC016 cell CM; ii, hASCs or HC016 cells; and iii, _OX_hASCs or _OX_HC016 cells. Four different experiments were performed in triplicate. Results were normalized to controls (HOGd, dotted line) and expressed as mean ± SD. * *p* < 0.05, compared with control, # *p* < 0.05, for pair comparison.

In the first case, in the presence of CM of hASCs or HC016 cells, at 48 h, the viability of _OX_HOGd recovered to levels similar to those of control cells (Figure 2A), and the intracellular ROS levels were significantly lower (Figure 2B). Regarding the indirect co-cultures, at 48 h, the recovery of the oligodendrocytes was evident in all cases. This recovery was more marked, however, when _OX_HOGd were co-cultured with the oxidized HC016 cells (_OX_HC016 cells) as indicated by significantly higher viability (1.4-fold higher, Figure 2A) and lower ROS levels (1.3-fold lower, Figure 2B) than when co-cultured with the oxidized hASCs (_OX_hASCs).

### 2.3. Antioxidant Capacity of HC016 Cells

**Figure 3:**
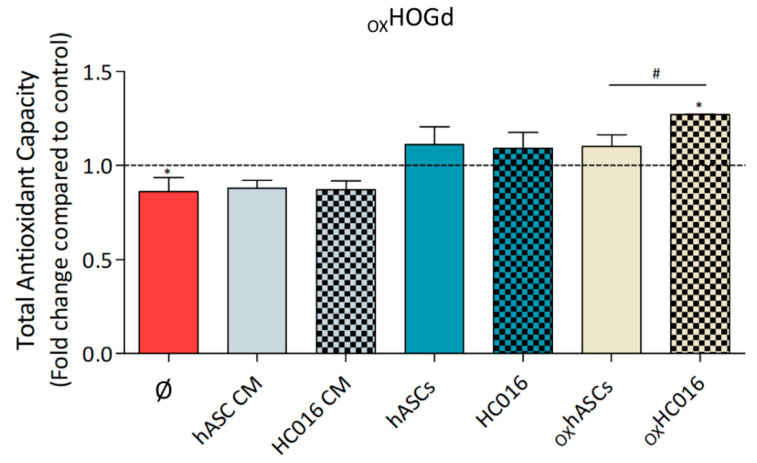
Total antioxidant capacity of the culture media of _OX_HOGd alone (Ø) or cultured with: i, hASC CM or HC016 cell CM; ii, hASCs or HC016 cells; and iii, _OX_hASCs or _OX_HC016 cells. Four different experiments were performed in triplicate. Results were normalized to controls (HOGd, dotted line) and expressed as mean ± SD. * *p* < 0.05, compared with control, # *p* < 0.05, for pair comparison.

Having evaluated the reparative effect of HC016 cells and their CM on _OX_HOGd, the antioxidant capacity of both cell types was analyzed. Secretion of proteins and small molecule antioxidants into the culture medium of _OX_HOGd alone or cultured with the different treatments was evaluated at 24 h after the H_2_O_2_ insult. Results showed that _OX_HOGd alone secreted a lower level of antioxidant molecules to the medium than control cells (1.2-fold lower). Whereas the media of _OX_HOGd cultured with hASC CM or HC016 cell CM, hASCs, HC016 or oxhASCs had values similar to those of the control, the medium of the co-culture of _OX_HOGd with oxHC016 cells showed a significantly higher level of antioxidant molecules (1.3-fold higher than in the control medium and 1.15-fold higher than the medium from the co-culture of _OX_HOGd with _OX_hASCs; Figure 3).

Since the analysis of the total antioxidant capacity in the co-cultures did not distinguish between the antioxidant response of HOGd or HC016 cells/hASCs, protein analysis of the two cells types was performed separately, determining the expression of different antioxidant proteins by western blot.

**Figure 4:**
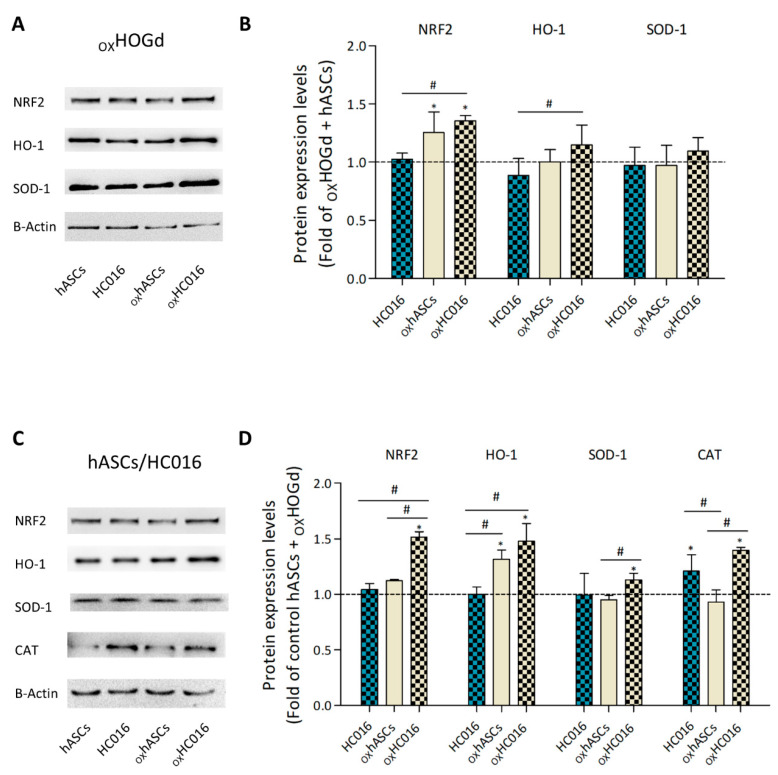
Expression of antioxidant proteins. (**A**) Expression and (**B**) quantification of Nrf2, HO-1 and SOD-1 in _OX_HOGd. HOGd were exposed to 0.25 mM H_2_O_2_ for 1 h, cultured with i, hASCs or HC016 cells and ii, _OX_hASCs or _OX_HC016 cells, and lysed 24 h after the H_2_O_2_ insult. Data were normalized to controls (_OX_HOGd + hASCs, dotted line). (**C**) Expression and (**D**) quantification of Nrf2, CAT, HO-1 and SOD-1 in hASCs or HC016 cells. hASCs, HC016 cells, _OX_hASCs or _OX_HC016 cells co-cultured with _OX_HOGd were lysed 24 h after the H_2_O_2_ insult. Data were normalized to controls (hASCs + _OX_HOGd, dotted line). At least three different experiments were performed, and results are expressed as mean ± SD. * *p* < 0.05, compared with control, # *p* < 0.05, for pair comparison.

First, the expression of Nrf2, HO-1, SOD-1 and CAT in HOGd was analyzed under the different conditions of interest (Figure 4A). The results revealed that when co-cultured with hASCs or HC016 cells, _OX_HOGd exhibited similar expression of Nrf2, HO-1 and SOD-1. In the case of _OX_HOGd co-cultured with _OX_hASCs or _OX_HC016 cells, Nrf2 expression was observed to be significantly higher (1.25 and 1.35-fold higher, respectively), differences between them not reaching significance. Concerning HO-1 and SOD-1, expression levels were not significantly different, though they were slightly higher in the cells co-cultured with oxHC016 cells (Figure 4B). CAT was not expressed in HOGd under any of the culture conditions.

Having evaluated the antioxidant protein expression in _OX_HOGd under the different conditions, Nrf2, CAT, HO-1 and SOD-1 expression were assessed in hASCs, HC016 cells, _OX_hASCs and _OX_HC016 cells co-cultured with _OX_HOGd (Figure 4C). After 24 h of co-culture, the level of nuclear Nrf2 expression in hASCs and HC016 cells was almost the same, whereas it was higher in _OX_hASCs and _OX_HC016 cells (1.12- and 1.51-fold higher, respectively), this difference being 1.35-fold larger in _OX_HC016 cells than _OX_hASCs. In contrast to what was observed in HOGd, CAT was expressed in the hASC and HC016 samples. In fact, it was significantly overexpressed in both HC016 cells and oxHC016 cells compared to the corresponding hASCs (1.2- and 1.4-fold increase, respectively). In the case of HO-1, hASCs and HC016 cells exhibited similar levels of expression, levels increasing when the cells were subjected to oxidative stress (1.3- and 1.5-fold increase, respectively). Last but not least, SOD-1 expression was also similar in hASCs and HC016 cells. When exposed to oxidative stress, the expression of SOD-1 increased in HC016 cells (1.3-fold increase), whereas the expression in hASCs remained similar to that in controls (Figure 4D).

## 3. Discussion

Oxidative stress plays a fundamental role in the pathophysiology of neurodegenerative disorders. It affects different cells of the CNS, but oligodendrocytes, glial cells with great impact on brain development and neuronal function, are the most vulnerable, mainly due to their low antioxidant defenses [19]. Over the years, many neuroprotective agents have been tested, seeking to protect cells against oxidative stress and prevent neuron loss, but clinical trials aiming to ameliorate brain injury have failed or been only partially successful [20]. Recently, cell therapy with MSCs has emerged as one of the most promising strategies for the treatment of neurodegenerative diseases. Their great antioxidant capacity [21], homing ability [22] and paracrine effects [23] make them good candidates for treating CNS pathologies associated with oxidative stress. Previous studies have demonstrated that MSCs have a protective effect on the CNS [24,25,26]; however, as mentioned before, their poor survival after transplantation is still a disadvantage, and to overcome this, MSC preconditioning has emerged as one of the main strategies to improve their survival and thereby their therapeutic effectiveness [27]. Additionally, many studies have focused on using MSC secretome [28,29]. MSCs are known to synthesize and secrete multiple bioactive factors that modulate the action of adjacent cells increasing angiogenesis, survival, migration, and differentiation; reducing apoptosis, fibrosis, and oxidative stress; restricting local inflammation, and adjusting immune responses [30,31,32]. Nonetheless, the fact that MSCs can dynamically change bioactive factor composition in response to various pathologies and environmental stimuli must be considered, because depending on the disease or environment in question, MSCs generate a specific molecular expression response through the activation of different pathways [33].

Here, we have investigated the therapeutic potential of HC016 cells and their CM to overcome the deleterious effect of oxidative stress in an oligodendroglial population. For this purpose, we selected the HOG cell line, which we differentiated towards oligodendrocyte-like cells (HOGd). Although primary cultures of human oligodendrocytes have been used in various studies, this approach is hampered by the limited availability of viable human brain tissue [34]. Hence, there was a need to develop a study model that allowed an unlimited supply of cells with characteristics similar to those of human oligodendrocytes. The protocol for the differentiation of HOG cells to oligodendrocyte-like cells worked well: HOGd exhibited extended branched processes and short membrane extensions, a decrease in proliferative activity, and an increase in the expression of oligodendrocyte markers, MBP, CNPase and MOG, which was consistent with already published reports [34,35,36,37]. Once the differentiation protocol had been established and a stable culture of HOGd obtained, we studied the effect of oxidative stress on these cells. Here, the oxidative stress induction model used was the administration of a single dose of 0.25 mM H_2_O_2_, one of the most important ROS involved in neuroinflammation and neurodegenerative disease [38]. This model allowed us to analyze, first, the effect of oxidation on cell viability and proliferation, as well as on the intracellular levels of ROS. As expected, the H_2_O_2_ insult caused a decrease in HOGd viability, which was in accordance with the significant increase observed in intracellular ROS levels.

Second, once the effect of oxidation on HOGd had been analyzed, we evaluated the therapeutic potential of HC016 cells, both through their CM and indirect co-culture. In the first case, in the presence of CM of both hASCs or HC016 cells, the population of HOGd exposed to oxidative stress recovered their viability to levels similar to those of the control cells, in addition to showing a significant reduction in intracellular ROS levels. The effect was even more pronounced when _OX_HOGd were indirectly co-cultured with hASCs or HC016 cells, and notably, at 48 h, _OX_HOGd cell viability actually exceeded that of the controls. Other authors have observed similar results with MSCs obtained from other tissues, indicating that MSCs exert a proliferative stimulus on other cell populations undergoing oxidative stress [39]. That is, although both strategies, CM and indirect co-culture, had reparative effects on _OX_HOGd, cell co-culture was associated with better restoration of _OX_HOGd function than CM. This may suggest that the presence of _OX_HOGd is needed to stimulate MSCs to produce the factors required for _OX_HOGd recovery. This hypothesis is supported by the observation of Fitzpatrick et al. that hepatocytes (HCs) cultured in medium previously conditioned by co-culturing HCs and MSCs demonstrated improved cell functionality, whereas HCs cultured in medium conditioned by MSCs alone were not affected [40]. Finally, among the different co-cultures, the _OX_HOGd co-cultured with HC016 cells also exposed to oxidative stress showed a better recovery with enhanced viability (1.4-fold that in controls) and lower ROS levels (1.3-fold those in controls). As previously demonstrated, the 0.25 mM H_2_O_2_ insult stimulated HC016 cell antioxidant response [18], and this seems to be beneficial for _OX_HOGd recovery.

Additionally, considering the lowering of ROS levels in _OX_HOGd after the treatments, we analyzed the concentration of proteins and small molecule antioxidants in the culture media of the _OX_HOGd alone or co-cultured with *i*, hASC CM or HC016 cell CM, *ii*, hASCs or HC016 cells, or *iii*, _OX_hASCs or _OX_HC016 cells. The results revealed that after 24 h, the co-culture of _OX_HOGd with HC016 cells exposed to oxidative stress secreted a significantly higher amount of antioxidant molecules to the medium, which was consistent with the previously observed decrease in intracellular ROS levels. These results, however, do not tell us whether the secretion of antioxidant molecules was due to _OX_HOGd or HC016 cells, and hence, we analyzed the expression of several antioxidant proteins in the different cell populations.

We first analyzed the expression of Nrf2, HO-1, SOD-1 and CAT in the oligodendrocytes under the various co-culture conditions tested. Although not significantly, levels of expression of Nrf2, HO-1 and SOD-1 were higher in _OX_HOGd co-cultured with _OX_HC016 cells than in those co-cultured with _OX_hASCs. Any increase in HO-1 is probably linked to an enhancement in Nrf2 expression since Nrf2 is recognized as a pivotal regulator of HO-1 induction in the nervous system. Nrf2-dependent activation of HO-1 is usually linked to a protective adaptation of neurons and glial cells, whereas the Nrf2-independent activation of HO-1 seems to exert neurotoxic effects. This is probably due to the ability of Nrf2 to also promote the transcription of other antioxidant and protective molecules [41].

The _OX_HOGd co-cultured with the HC016 cells showed the most evident recovery 24 h after the insult with H_2_O_2_, and it is conceivable that this was related to the activation of the Nrf2/HO-1 signaling pathway. In accordance with this, other authors have already reported the Nrf2/HO-1 signaling pathway role in reducing cytotoxicity and oxidative stress induced by LPS in oligodendrocytes [42]. Moreover, SOD-1 expression was also slightly increased in _OX_HOGd co-cultured with oxHC016 cells, which is in agreement with the Nrf2 outcome. Several authors relate SOD-1 expression to that of Nrf2 [43], and with ROS reductions, since it has been observed that mature oligodendrocytes overexpressing SOD-1 are more resistant to oxidative stress, enhancing their survival [44]. Ultimately, CAT was not expressed in _OX_HOGd under any of the culture conditions, in line with data obtained by Baud et al., who stated that rat oligodendrocytes subjected to a 1 h insult of H_2_O_2_ inactivated the catalase activity [45].

Secondly, regarding the hASCs or HC016 cells co-cultured with the _OX_HOGd, a significant increase was observed in the expression of Nrf2, HO-1, and SOD-1 in the _OX_HC016 cells compared to that in _OX_hASCs. This result is in line with that obtained by analyzing the total antioxidant capacity of the medium from _OX_HOGd co-cultured with _OX_HC016 cells and is similar to the observed expression of the antioxidant enzymes in _OX_HOGd, which suggests that exposure to soluble factors secreted by activated HC016 cells upregulates the expression of antioxidant enzymes in _OX_HOGd. In accordance with this, several authors have already linked an increase in the expression of antioxidant enzymes in MSCs with the recovery of damaged tissue. For example, Li et al. reported that overexpression of antioxidants, specifically HO-1, could enhance the ability of MSCs to protect or repair retinal cells [46].

Additionally, in contrast to what was observed in the _OX_HOGd, CAT did express in hASCs and HC016 cells. In fact, it was significantly overexpressed in both HC016 cells and _OX_HC016 cells compared to the corresponding hASCs (1.2 and 1.4-fold higher, respectively). Since the higher CAT expression in HC016 cells, in both standard or oxidative stress conditions, coincided with the lower intracellular ROS levels in _OX_HOGd, we hypothesize that CAT also played an important role in reducing ROS levels in this cell population because it is an efficient scavenger of H_2_O_2_ when it is present in high concentrations [47].

In summary, this study shows that adaptation to oxidative stress is a fundamental advantage for the therapeutic capacity of hASCs. Our results suggest that HC016 cell exposure to H_2_O_2_ stimulates their antioxidant response, which in turn appears to promote the antioxidant response of damaged oligodendrocytes, reducing their ROS levels and increasing their viability. Moreover, HC016 cells have also shown a more efficient response to _OX_HOGd rescue signals than hASCs. These findings taken together suggest that HC016 cells are good candidates for overcoming oligodendroglial oxidative stress-induced damage.

## 4. Materials and Methods 

### 4.1. Cell Culture

Human adipose-derived stem cells (hASCs) were kindly donated by Histocell S.L. (Science and Technology Park of Bizkaia, Derio, Spain). These cells were maintained in DMEM Glutamax™ (Dulbecco’s Modified Eagle Medium, Gibco, Paisley, UK) supplemented with gentamicin (1 µL/mL, Sigma-Aldrich, St. Louis, MO, USA) and 10% heat-inactivated fetal bovine serum (FBS, Biochrom, Berlin, Germany), and incubated at 37 °C, in a humidified atmosphere containing 5% CO_2_. Cells up to passage 4 were used in this study.

Human oligodendroglioma (HOG) was kindly donated by Dr. Glyn Dawson (University of Chicago, IL, USA). HOG cells were maintained in DMEM (Dulbecco’s Modified Eagle Medium, Gibco, Paisley, UK) supplemented with gentamicin (1 µL/mL), L-glutamine (Gibco, Paisley, UK), sodium pyruvate (Gibco, Paisley, UK) and a 10% of heat-inactivated FBS, and incubated at 37 °C in a saturated humidity atmosphere containing 5% CO_2_. Cells were used until passage 15.

### 4.2. HOG Differentiation

**Figure 5:**
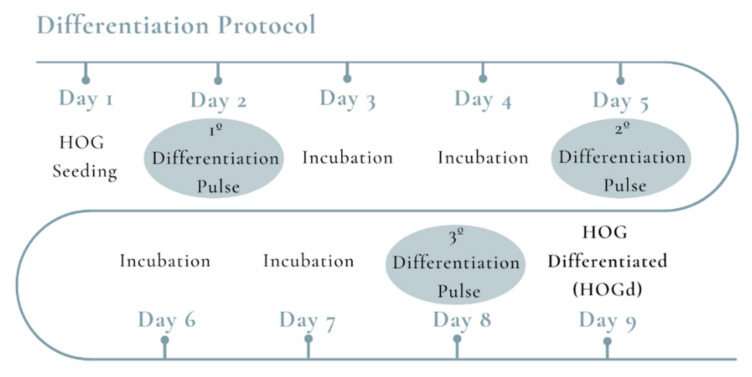
Chronogram of HOG cell line differentiation.

In this study, we differentiated HOG cell line to oligodendrocyte-like cells (HOGd). For their differentiation, the culture vessels were coated with poly-D-lysine (PDL, Sigma-Aldrich, St. Louis, MO, USA) and the HOG cell line was cultured at a 22 × 10^4^ cells/cm^2^ in complete medium for 24 h. Then, complete medium was replaced by differentiation medium (DM) containing high glucose (4.5 g/L) DMEM with N2 supplement (Life Technologies, Eugene, OR, USA), 30 nM triiodothyronine (T3, Sigma-Aldrich, St. Louis, MO, USA) and 0.05% FBS, and cells were cultured for 9 days with three medium refreshments (Figure 5).

### 4.3. Immunofluorescence Assay

For assessing HOG cell differentiation, immunofluorescence assays were performed to detect oligodendrocyte-specific markers (MBP, CNPase and MOG). For this experiment, cells were seeded and differentiated in µ-Slide 8 wells (Ibidi GmbH, Martinsried, Germany). Cells were fixed with 4% paraformaldehyde (PFA, PanReac AppliChem, Barcelona, Spain) for 15 min at RT and then incubated with PBS, containing 3% BSA and 0.1% Triton X-100, for 30 min at RT to permeabilize the cells and block non-specific protein-protein interactions. Subsequently, cells were incubated overnight at 4 °C with primary antibodies against MBP, CNPase and MOG (1:100, Genetex, Irvine, CA, USA). After washing three times with 1x PBS, cells were incubated with a secondary antibody (Goat anti-Rabbit IgG Alexa 488, 1:2000, Invitrogen, Eugene, OR, USA) for 1 h at RT in the dark. Nuclei were counterstained with Hoechst 33,342 (Abcam, Cambridge, UK). Finally, cells were washed three more times and observed under a Zeiss LSM800 confocal microscope (Carl Zeiss Inc, Chicago, IL, USA) using x20 objective.

### 4.4. H_2_O_2_-Preconditioning of hASCs

Long-term exposure to a low concentration of H_2_O_2_ (PanReac AppliChem, Barcelona, Spain) was used to obtain H_2_O_2_-preconditioned hASCs (HC016 cells). Briefly, hASCs were exposed to 10 µM of H_2_O_2_ for 7 days, with replenishment of oxidative culture medium twice during the preconditioning protocol (see details in HC016 patent; WO/2013/004859, 2013). Non-preconditioned hASCs were cultured in parallel for the same number of passages. Once the preconditioning process had been completed, HC016 cells and hASCs were seeded at high density and incubated at 37 °C in a humidified atmosphere containing 5% CO_2_ for 18–20 h in complete medium, until use in experiments.

### 4.5. Oxidative Stress Induction

Oxidative stress conditions were achieved using a dose of 0.25 mM H_2_O_2_ based on the results of previous experiments performed in our group. For the different experiments, HOG cells were seeded onto 24- (Sarstedt, Nümbrecht, Germany) or 6-well plates (Corning, Tewksbury, MA, USA). Once they reached 80% confluency, HOG cells were differentiated and then exposed to a 0.25 mM H_2_O_2_ insult for 1 h (_OX_HOGd) in DMEM FBS free at 37 °C, in a humidified 5% CO_2_ atmosphere. After that period, media were replaced with fresh DMEM FBS free.

### 4.6. Treatments

**Figure 6:**
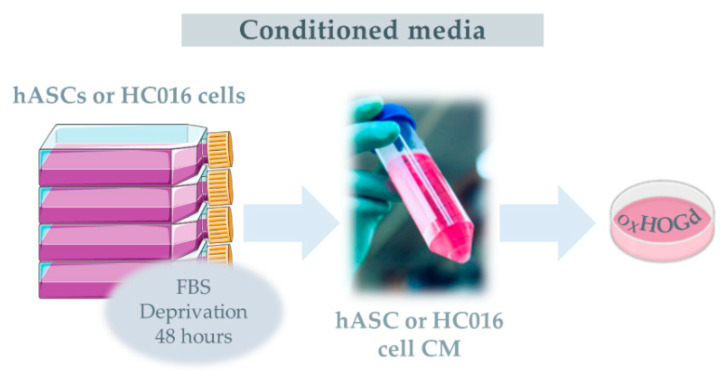

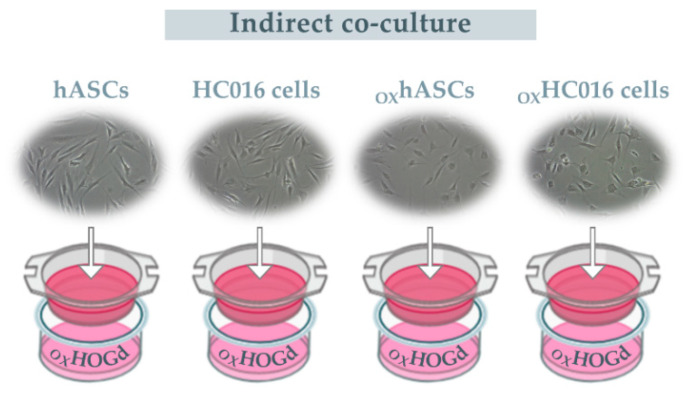
Visual representation of hASC or HC016 cell CM and indirect co-culture treatments for _OX_HOGd recovery.

To evaluate the therapeutic potential of HC016 cells, we used two different treatment strategies. The first one was the culture of damaged HOGd (_OX_HOGd) with hASC or HC016 cell conditioned media (CM). On the other hand, the second therapeutic strategy was the indirect co-culture of _OX_HOGd and hASCs or HC016 cells which had or had not been exposed to H_2_O_2_ for 1 h (Figure 6).

To obtain hASC or HC016 cell CM, cells were grown in T175 (Sarstedt, Nümbrecht, Germany) or T636 (Corning, Tewksbury, MA, USA) tissue culture flasks to 80% confluence, washed three times with 1x PBS and incubated for 48 h in DMEM Glutamax™ FBS free. Then, the CM was centrifuged for 5 min at 250 g, filtered with a 0.2 µm filter (Filtropur S 0.2, Sarstedt Nümbrecht, Germany) and stored at −20 °C until use.

For the indirect co-culture treatment, hASCs or HC016 cells were seeded onto cell culture inserts with 3-µm pore size (Nunc™, Life Technologies, Eugene, OR, USA) in 24- or 6-well plates at a concentration of 3 × 10^4^ cells/cm^2^. Cells were incubated in DMEM Glutamax™ with 10% FBS at 37 °C in a saturated humidity atmosphere containing 5% CO_2_ to allow cell adhesion (2–4 h). Once adhered to the insert, the complete media were removed and hASCs or HC016 cells were indirectly co-cultured with _OX_HOGd, with or without exposing them to 1 h oxidative stress insult.

### 4.7. Proliferation Assay

Proliferation of _OX_HOGd was assessed by counting viable cells at 24 and 48 h with PrestoBlue^®^ (Invitrogen, Eugene, OR, USA), a resazurin-based solution that functions as a colorimetric cell viability indicator. At each time point, treated _OX_HOGd cultured in 24-well plates were incubated with PrestoBlue^®^ reagent following the manufacturer instructions. The optical density (OD) was measured at λ 570 nm in a spectrophotometer. Results were normalized to controls (HOGd) and expressed as the means ± SD of four different experiments performed in triplicate.

### 4.8. Measurement of ROS

Intracellular levels of ROS in _OX_HOGd were detected using an H2-DFC-DA probe (Invitrogen, Eugene, OR, USA) at 24 and 48 h after the various treatments used. H2-DFC-DA is a chemically reduced form of fluorescein that upon cleavage of the acetate groups by intracellular esterases and ROS is converted to highly fluorescent DCF. This probe was added to the cells at a final concentration of 10 µM, for 30 min at 37 °C. After this period, the fluorescent probe was removed and cells were washed with 1x PBS. Finally, intracellular ROS accumulation was measured in a microplate reader (λex 492–495 nm; λem 517–527 nm). Results were normalized to controls (HOGd) and represented as the means ± SD of four different experiments performed in triplicate.

### 4.9. Total Antioxidant Capacity Assay

A Total Antioxidant Capacity Assay Kit (Abcam, Cambridge, UK) was used to determine the capability of _OX_HOGd cultured with the different treatments to counteract ROS. The kit analyzes the concentration of small molecule antioxidants and proteins in the culture media. Briefly, after 24 h of treatment, a Trolox standard curve (4–20 nmol/well) was prepared and media of the different culture conditions were collected and diluted 1:2 in ddH_2_O. Diluted fresh samples (100 µL) were mixed with Cu^2+^ working solution (100 µL) in a 96-well plate and incubated at RT for 90 min protected from light. Afterwards, the OD was measured at λ 570 nm in a spectrophotometer. Results were normalized to controls (HOGd) and reported as the average of four different experiments (mean ± SD) performed in duplicate.

### 4.10. Western Blot Analysis

To determine the expression of proteins of interest, cells were seeded onto 6-well plates or onto inserts. After the HOG differentiation and 24 h after the treatments, cells were lysed in 1× Laemmli buffer (Sigma-Aldrich, St. Louis, MO, USA) and homogenized by sonication. Protein was quantified using a turbidimetric method based on TCA. Then, quantified protein lysates were boiled for 5 min for their denaturation. Denatured samples were loaded onto 10% SDS-PAGE gels and resolved by electrophoresis, using an SDS running buffer (25 mM Tris, 0.2 mM glycine, 0.1% SDS (*w*/*v*)) at 90 V, for 90 min in a Mini-Protean^®^ 3 Cell. Resolved proteins were transferred onto nitrocellulose membranes at 380 mA, for 180 min at 4 °C. Subsequently, nitrocellulose membranes were stained with Ponceau S solution to visualize resolved proteins. Ponceau S solution was removed from the membranes by washing them with TBS-T (20 mM Tris, pH 7.5, 500 mM NaCl, 0.1% Triton X-100 (*v*/*v*)). Afterwards, membranes were blocked for 1 h at RT in blocking buffer (5% skimmed milk in TBS-T) and then incubated overnight on a shaker at 4 °C, with the following primary antibodies: MBP (1:500), CNPase (1:1000), MOG (1:500), Nrf2 (1:1000), SOD-1 (1:1000), HO-1 (1:1000), GPx1 (1:1000), CAT (1:1000, Genetex, Irvine, CA, USA) and β-Actin (1:5000, EMD Millipore, Darmstadt, Germany). Subsequently, membranes were washed three times with TBS-T for 10 min, incubated with secondary antibody (Goat anti-Rabbit IgG, 1:1000, Thermo Fisher Scientific, Waltham, MA, USA) for 1 h at RT in blocking buffer and washed as before. Finally, membranes were visualized using SuperSignal West Pico PLUS Chemiluminescent Substrate (Thermo Fisher Scientific, Waltham, MA, USA). Images were acquired with the gel documentation system, G: Box Chemi (Syngene, Cambridge, UK) and densitometry was performed with the ImageJ software (Madison, WI, USA). Densitometry values were then normalized to those of their corresponding loading controls. Resulting data were normalized to each control and results were reported as the average of at least three different experiments (mean ± SD).

### 4.11. Statistical Analysis

The number of samples analyzed was described in each experiment. All data are shown as mean ± SD. Statistical analysis was performed using GraphPad Prism statistical software (version 5.0; GraphPad Software, San Diego, CA, USA). Significance was assessed using analysis of variance followed by Bonferroni’s post hoc test and t-tests, as appropriate. Statistical differences were considered significant where *p* < 0.05. All the images shown in this manuscript represent the data obtained in, at least, three independent experiments with similar results.

## Abbreviations

MSCs	mesenchymal stem cells
hASCs	human adipose-derived stem cells
HC016	hASCs preconditioned with low doses of H_2_O_2_
HOGd	oligodendrocyte-like cells
